# DYT-TOR1A genotype alters extracellular vesicle composition in murine cell model and shows potential for biomarker discovery

**DOI:** 10.3389/dyst.2023.11053

**Published:** 2023-02-15

**Authors:** Connor S. King, Zachary F. Caffall, Erik J. Soderblom, Nicole Calakos

**Affiliations:** 1 Department of Neurology, Duke University Medical Center, Durham, NC, United States; 2 Department of Cell Biology, Duke University Medical Center, Durham, NC, United States; 3 Proteomics and Metabolomics Shared Resource, Department of Biostatistics and Bioinformatics, Duke University Medical Center, Durham, NC, United States; 4 Department of Neurobiology, Duke University Medical Center, Durham, NC, United States; 5 Duke Institute for Brain Sciences, Duke University, Durham, NC, United States

**Keywords:** dystonia, biomarkers, proteomics, DYT1, extracellular vesicles, ritonavir

## Abstract

**Introduction:**

Biomarkers that can be used to identify patient subgroups with shared pathophysiology and/or that can be used as pharmacodynamic readouts of disease state are valuable assets for successful clinical trial design. In translational research for brain diseases, extracellular vesicles (EVs) have become a high-priority target for biomarker discovery because of their ubiquity in peripheral biofluids and potential to indicate brain state.

**Materials and methods:**

Here, we applied unbiased quantitative proteomics of EVs isolated from DYT-TOR1A knockin mouse embryonic fibroblasts and littermate controls to discover candidates for protein biomarkers. We further examined the response of genotype perturbations to drug treatment conditions to determine their pharmacodynamic properties.

**Results:**

We found that many DYT-TOR1A MEF EV differences were significantly corrected by ritonavir, a drug recently shown to correct DYT-TOR1A phenotypes in cell and mouse disease models. We also used tool compounds to explore the effect of the integrated stress response (ISR), which regulates protein synthesis and is implicated in dystonia pathogenesis. Integrated stress response inhibition in WT cells partially phenocopied the effects of DYT-TOR1A on EV proteome composition, and ISR potentiation in DYT-TOR1A caused changes that paralleled ritonavir treatment.

**Conclusion:**

These results collectively show that DYT-TOR1A genotype alters EV protein composition, and these changes can be dynamically modulated by a candidate therapeutic drug and ISR activity state. These mouse model findings provide proof-of-concept that EVs may be a useful source of biomarkers in human populations and further suggest specific homologs to evaluate in cross-species validation.

## Introduction

Dystonia is a movement disorder characterized by sustained muscle contractions with abnormal twisting movements (1). DYT-TOR1A is a rare inherited dystonia caused by a mutation in *TOR1A* (n. delGAG, p. ΔE) leading to a childhood-onset form of the disease that often involves most of the body (e.g., early-onset, generalized dystonia) (2). Currently, there is substantial unmet clinical need for DYT-TOR1A dystonia treatment. Oral medications are limited by narrow therapeutic windows and side effects, typically leaving deep brain stimulation surgery as the major alternative treatment option (3). To fill these treatment gaps, drug discovery efforts are underway to identify highly effective, well tolerated, and orally bioavailable small molecules. We have previously demonstrated that ritonavir, an HIV protease inhibitor, rescues diverse disease phenotypes in DYT-TOR1A preclinical models (4). However, translating effective treatments from the bench into the clinic is especially difficult for neurological diseases, which have a below average success rate in all clinical trial phases compared to other body systems (5, 6). One strategy to improve clinical trial design is identifying and measuring biomarkers before and during the treatment intervention. Biomarkers have multiple classifications depending on their clinical context of use. These include predictive biomarkers, which can be used to stratify patient subpopulations and enrich recruitment for subjects most likely to respond to the given intervention, and pharmacodynamic/response biomarkers, which track physiological changes throughout treatment to assess successful target engagement (7, 8). The incorporation of such biomarkers into clinical trials can double the likelihood of success from Phase I through final regulatory approval (5). Thus, biomarkers are a valuable asset, especially for rare and neurological diseases.

Peripheral biofluids are an easily accessible source of molecular biomarkers, such as proteins, lipids, and miRNAs (9–11). In a variety of clinical settings, these ‘liquid biopsies’ are now performed routinely to measure disease processes that often occur quite distal to the puncture collection site (9, 10). For example, tumor-derived DNA circulating in blood can reveal mutations that predict response to particular chemotherapies (12), and hemoglobin A1C in diabetes is both a diagnostic biomarker during initial screening and a pharmacodynamic/response biomarker for monitoring blood glucose after treatment (7). However, in diseases of the CNS like dystonia, peripheral biomarkers for brain state are more challenging to isolate because of the blood brain barrier (BBB). Extracellular vesicles (EVs) have been found to be a promising source for CNS disease biomarkers, since they can cross the BBB and carry protein and RNA cargo secreted by brain cells (13). As one example, neurofilament light chain in plasma EVs has been studied in X-linked dystonia-parkinsonism and other neurodegenerative diseases as a biomarker for brain axonal degeneration (14, 15). Thus, EVs are often considered to provide a view into the physiological state of their cells of origin.

In this study, we first sought to determine whether the DYT-TOR1A genotype altered EV composition, a finding that would open the possibility to use EVs as biomarkers in this disease. We focused on obtaining proof-of-concept in cell lines which also secrete EVs because DYT-TOR1A is a rare genetic disease with geographically isolated human subject populations (16, 17). While we considered DYT-TOR1A patient-derived cell lines (14, 18), we chose to use murine embryonic fibroblasts (MEFs) derived from the Tor1a^ΔGAG/+^ knockin mouse model of DYT-TOR1A (19) because it has construct validity and also provides a uniform genetic background to reduce variability in a proof-of-concept experiment. We examined the effects of DYT-TOR1A on EV protein composition using quantitative LC-MS/MS proteomics. Once putative genotype-modified candidates were identified, we next explored their behavior in response to pharmacological manipulations: therapeutic treatment with a candidate dystonia drug, ritonavir, and modulation of a conserved signaling pathway perturbed in multiple dystonias, the integrated stress response (ISR) (20). Lastly, we combined our experimental observations with pragmatic criteria for ideal clinical biomarkers to put forth candidates with the highest potential for future tests of DYT-TOR1A EV biomarkers in human subjects.

## Results

### DYT-TOR1A MEF EVs show altered protein composition

**FIGURE 1:**
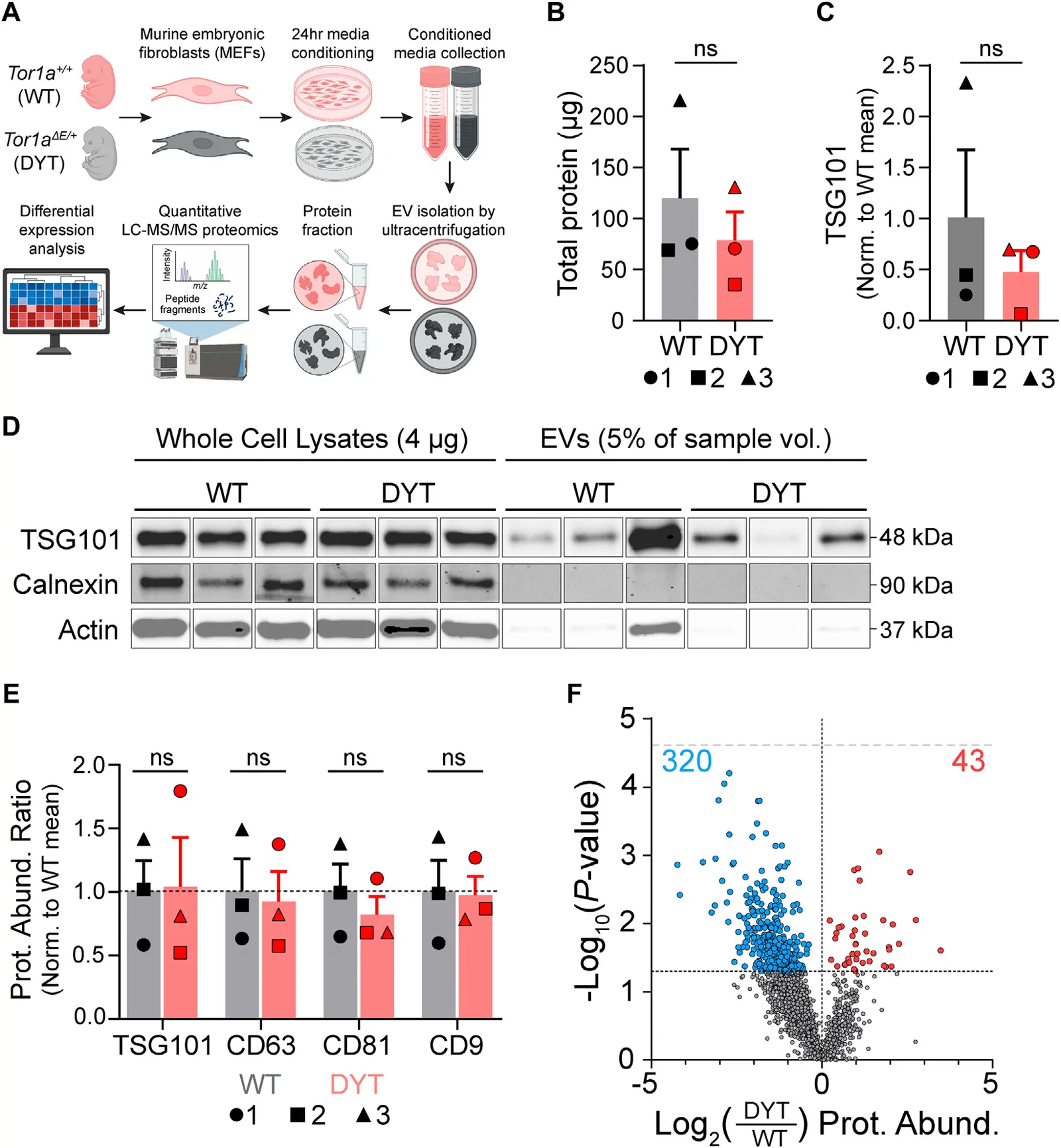
Quantitative proteomics shows DYT-TOR1A-dependent changes in protein composition of EVs isolated from murine embryonic fibroblasts. **(A)** Experimental workflow schematic. **(B)** Total protein in EV samples quantified by BCA. 1, 2, and 3 indicate specific biological replicates. **(C)** Quantification of TSG101 from Western blot in **(D)** normalized to the mean WT abundance. 1, 2, and 3 indicate specific biological replicates. **(D)** Western blot of whole cell lysates (4 μg protein) and EV samples (5% of total EV sample by volume) for TSG101 (EV marker), calnexin (ER marker), and actin. See Supplementary Figure S1 for complete blot images. **(E)** Mass spectrometry protein abundances (Prot. Abund.) of EV markers normalized to WT mean abundance. 1, 2, and 3 indicate specific biological replicates. Significance testing in **(B,C,E)** by unpaired Student’s t tests. **(F)** Volcano plot comparing protein abundances of 1974 detected EV proteins between DYT-TOR1A and WT. Symbol color of data points indicate proteins significantly (*p* ≤ 0.05) more (red dots) or less (blue dots) abundant in DYT-TOR1A relative to WT. Lower horizontal dashed line indicates *p*-value of 0.05 threshold. Upper gray horizontal dashed line indicates Bonferroni-adjusted *p*-value threshold of 2.5e-5. Differences in abundance are represented as fold change (using log_2_ transformation) and *p*-value is calculated by unpaired t-test for each protein (*n* = 3 biological replicates).

Immortalized murine embryonic fibroblast (MEF) cell lines were prepared from heterozygous knockin mice bearing the DYT-TOR1A mutation (Tor1a^ΔGAG/+^ genotype hereafter abbreviated as DYT-TOR1A or DYT) (19) and wildtype (WT) littermate embryos according to standard methodology (Methods). Three independent cell lines for each genotype were used. Genotype and all drug treatment conditions were tested in a blinded experimental design and in parallel by splitting the parental cell line flask into separate flasks for each condition. EVs produced during the 24-h period following media exchange with an EV-depleted media were isolated from the conditioned media by ultracentrifugation (21). Protein was isolated from the resultant EV pellet. DYT-TOR1A did not significantly modify recovery of total protein or amount of the constitutive EV marker, TSG101 (Figures 1B, C). Specific EV enrichment was confirmed by Western blot for TSG101 compared to non-EV markers (calnexin, actin) (Figures 1C, D) (22). Samples were then subjected to unbiased, quantitative LC-MS/MS proteomics analysis. Quantitative proteomic measurements also demonstrated that EV protein abundances of classic EV markers (TSG101 and the tetraspanins CD9, CD81, and CD63) were not modified by genotype (Figure 1E).

We characterized the EV proteome to identify genotype-dependent changes in EV protein abundances between DYT-TOR1A and WT EV samples. Following alignment of peptide signals to unique identifying peptides (UIPs) and removal of proteins with fewer than two detected UIPs, 1974 proteins were detected across all cell lines. Using a Bonferroni-adjusted *p*-value threshold for multiple hypothesis testing (*p* < 2.5e-5) (23), no significant genotype effects were identified. We next used this discovery dataset to identify putative DYT biomarkers for testing in follow-on experiments. Using an uncorrected *p*-value cutoff of less than 0.05, we identified 363 of 1974 proteins with significantly different abundances in DYT-TOR1A versus WT EVs (Figure 1F). This differential subset of 363 is more than 3.5 times larger than would be predicted by chance (e.g., 99 proteins from the total of 1974, based on the expected proportion *α* = 0.05).

We further noted that among the 363 differential proteins, there was an asymmetric distribution of genotype effects. The DYT-TOR1A effects showed a bias towards decreased abundances, with 320 proteins being significantly less abundant compared to only 43 being more abundant in DYT-TOR1A relative to WT (two-tailed binomial sign test, *p* < 0.0001). This skewed distribution was also maintained across all EV proteins (1491 less, 483 more; two-tailed binomial sign test, *p* < 0.0001).

We therefore considered technical reasons that could artifactually cause such a distribution bias, e.g., lower EV yields and/or detection thresholds not being met preferentially in DYT samples. As Figure 1E demonstrates, there were no significant genotype-dependent differences in abundance of EV constituents detected in the LC-MS/MS data. Secondly, when a protein is not detected in a sample, an imputed value is given as described in Methods prior to sample loading normalization. We therefore examined whether the DYT genotype effects came preferentially from proteins with multiple imputed values. Instead, we observed that hits were distributed proportionally across proteins with 0, 1, 2 or 3 imputed values and the vast majority of hits came from proteins with no imputed values (Supplementary Figure S2). These observations rule out LC-MS/MS detection thresholds as a systematic confound. In summary, we have performed proteomic analysis of MEF culture-derived EV preparations and identify 363 candidate proteins for DYT-TOR1A genotype biomarkers.

### Ritonavir shows corrective effects on DYT-TOR1A EV protein composition

Recent studies have shown corrective effects of the HIV protease inhibitor ritonavir on cell and brain phenotypes in DYT-TOR1A preclinical models (4). For translation to human clinical trials, it is desirable to have pharmacodynamic biomarkers to aid early dose-finding studies and to assess target engagement (7, 8, 11). To explore the potential for the DYT-TOR1A genotype-associated EV changes that we identified to be used as pharmacodynamic biomarkers of disease state, we exposed DYT-TOR1A MEF cultures to 20 μM ritonavir throughout the 24 h of media conditioning preceding EV isolation. EV protein fractions were analyzed by quantitative LC-MS/MS proteomics performed in the same batch run as all conditions reported in this study.

**FIGURE 2:**
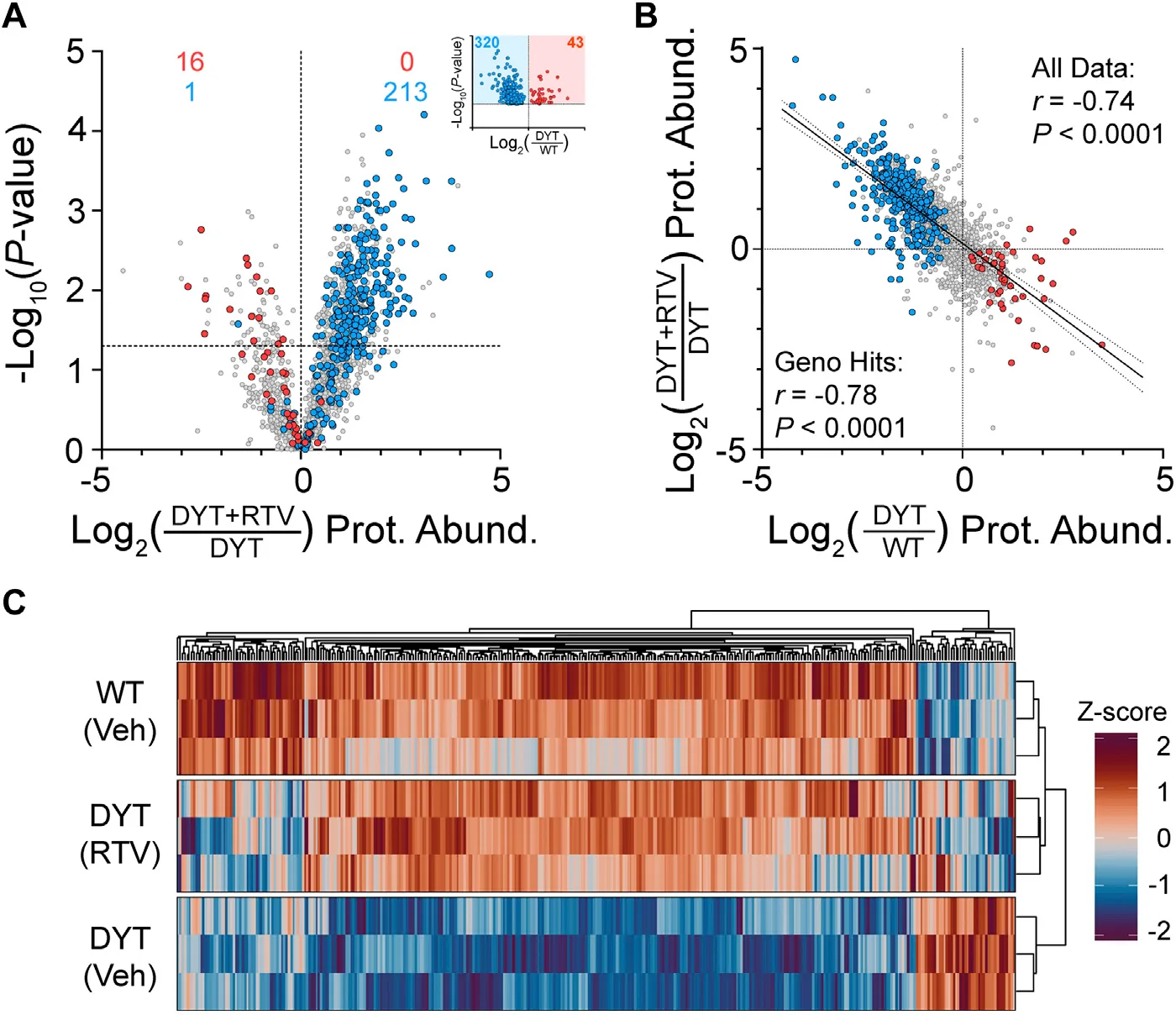
Effects of ritonavir treatment on protein composition of EVs isolated from DYT-TOR1A MEF cultures. **(A)** Volcano plot comparing protein abundances (Prot. Abund.) between EVs isolated from DYT-TOR1A MEF cultures treated with ritonavir (DYT+RTV) vs. vehicle (DYT). For **(A,C)**, differences in protein abundance are represented as fold change (using log_2_ transform) and *p*-value is calculated by unpaired t-test for each protein (*n* = 3 biological replicates). Horizontal dashed line indicates uncorrected *p*-value of 0.05. Color-coded data points indicate the original genotype disrupted proteins from Figure 1F with coloring showing the protein’s genotype effect (red being increased and blue being decreased in DYT/WT. Inset shows genotype results from Figure 1F). **(B)** Comparison of genotype (DYT/WT) and ritonavir (DYT+RTV/DYT) effects on protein abundances (log_2_ transformed). **(C)** Hierarchical clustering heatmap of WT, DYT, and DYT+RTV protein abundances for proteins significantly different in DYT relative to WT (*n* = 363).

Of the subset of 363 proteins significantly disrupted by DYT-TOR1A genotype basally, we found that >60% (230/363) had significant changes in abundance following ritonavir treatment at a threshold of *p* ≤ 0.05. This number of hits is 12 times greater than would be predicted by chance if ritonavir had no true effect on the genotype-dependent hits (18.15 proteins by *α* = 0.05). We further noticed that when examining the behavior of the 363 putative DYT biomarkers independent of *p*-value, the overwhelming majority of proteins showed ritonavir effects on protein abundance that were in the corrective direction (344/363) (Figure 2A). The putative DYT biomarker subset of proteins also showed strong and inverse correlations between genotype and ritonavir effects (Pearson *r* = −0.78, *p* < 0.0001) (Figure 2B). Noticing the very large number of proteins modified by ritonavir, we further examined the relationship between DYT genotype disruptions and DYT+RTV effects across the entire proteome and found that the strong inverse correlation was maintained (*n* = 1974, Pearson *r* = −0.74, *p* < 0.0001) (Figure 2B). Proteome-wide, ritonavir significantly modified 29% of the DYT EV proteome (uncorrected *p* ≤ 0.05, 582/1974) in a direction that was opposite to the genotype effect, with an asymmetric distribution toward increasing abundances for both significant and non-significant abundance changes (two-tailed binomial sign test: 508/582, with log_2_ fold change >0, *p* < 0.0001; 1372/1974 with log_2_ fold change >0, *p* < 0.0001). Lastly, we deployed hierarchical clustering to evaluate ritonavir’s effects on the putative DYT-TOR1A genotype biomarker proteins (*n* = 363). This analysis showed that ritonavir-treated DYT-TOR1A EV samples clustered more closely with WT than DYT-TOR1A samples (Figure 2C).

In summary, DYT-TOR1A genotype disruptions of EV protein composition show potential as pharmacodynamic markers of disease state. Ritonavir treatment acutely modified a substantial fraction of DYT-TOR1A genotype-dependent protein disruptions (95%) and caused dendrogram clustering of the EV proteome to become more closely related to WT samples than the DYT-TOR1A genotype.

### Influence of the integrated stress response pathway on EV composition in WT and DYT-TOR1A

DYT-TOR1A and other dystonias show dysfunction in a biochemical pathway, the integrated stress response (ISR), that has wide-reaching effects on the proteome because it regulates global protein synthesis (20). This prompted us to ask how the broad EV compositional differences we observed in the previous 2 experiments were related to ISR pathway effects.

We used ISR tool compounds to modify ISR activity. Our prior studies established the corrective directionality of the eIF2α phosphatase inhibitor salubrinal in DYT-TOR1A cell and mouse model phenotypes and sufficiency of the ISR inhibitor ISRIB to mimic DYT-TOR1A phenotypes (4, 20, 24). We therefore hypothesized that ISRIB-induced EV composition changes in WT MEF EVs would reproduce DYT-TOR1A genotype differences that were related to ISR dysregulation and that salubrinal treatment of DYT-TOR1A MEF EVs would cause normalizing shifts in genotype differences if they were related to ISR dysregulation.

**FIGURE 3:**
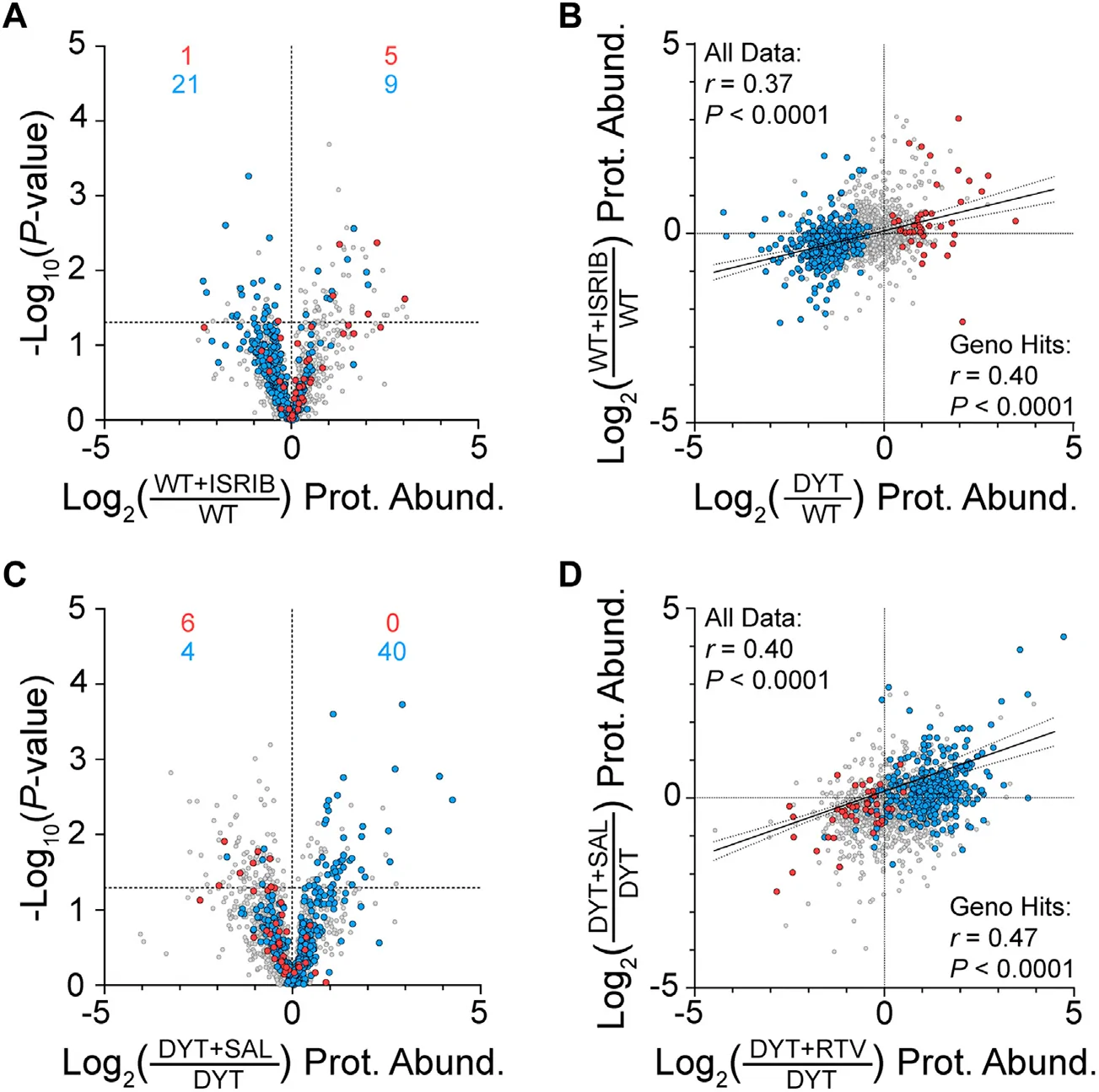
Effects of ISR tool compounds on MEF EV protein composition. **(A)** Volcano plot shows ISRIB effects on protein abundances in WT MEFs. For **(A–D)**, color-coded data points indicate proteins significantly increased (red) or decreased (blue) in DYT-TOR1A MEF EVs compared to WT experiment shown in Figure 1F. Differences in protein abundance are represented as fold change (using log_2_ transform) and *p*-value is calculated by unpaired t-test for each protein (*n* = 3 biological replicates). Horizontal dashed line indicates *p*-value of 0.05. **(B)** Correlation of protein abundance fold changes between DYT genotype effects (DYT/WT) and ISRIB effects (WT+ISRIB/WT). **(C)** Volcano plot showing the effect of salubrinal treatment of DYT MEFs (DYT+SAL) on EV protein abundances compared to vehicle control (DYT). **(D)** Correlation between ritonavir and salubrinal treatment effects on protein abundances in DYT MEF EVs.

WT MEFs were treated with 50 nM ISRIB to inhibit ISR pathway output for 24 h prior to EV harvest from the conditioned media. ISRIB treatment of WT cells disrupted fewer proteins at the statistical threshold of *p* ≤ 0.05 than were observed between DYT and WT samples (103/1974 (5%) vs. 363/1974 (18%)) and only 7% of the genotype-disrupted proteins (26/363) were reproduced by ISRIB at the statistical threshold (*p* ≤ 0.05). However, an examination of proteome-wide effects independent of *p*-value thresholds showed protein abundance directionality (greater or lesser) to be non-randomly distributed (Fisher’s exact test, *p* < 0.0001) and in a directionality similar to the DYT genotype effects (Figure 3A). A Pearson’s correlation analysis showed a positive correlation between DYT genotype effects and ISRIB effects, supporting the hypothesis that ISRIB treatment of WT cells mimics DYT genotype effects (Pearson *r* = 0.40, *p* < 0.0001)(Figure 3B). Interestingly, as was observed with ritonavir effects, this correlation was also maintained when the entire proteome was evaluated (Pearson *r* = 0.37, *p* < 0.0001).

To augment ISR activity in DYT-TOR1A MEFs, cell cultures were treated with 20 μM salubrinal during the 24 h conditioning period prior to EV harvest from the media. Salubrinal is a specific inhibitor of eIF2α phosphatases, CReP and GADD34 (25). Salubrinal treatment of DYT samples significantly modified 9% of the total proteins (169/1974) and caused significant corrective effects on 13% of DYT disrupted proteins (46/363) (Figure 3C). Like ISRIB, secondary analyses of effects independent of *p*-value thresholds showed that DYT disrupted proteins were not randomly distributed (Fisher’s exact test, *p* < 0.0001) and showed directionality biases supporting the hypothesis that salubrinal has corrective effects on DYT disruptions (Figure 3C). Lastly, we examined the concordance of drug effects between salubrinal and ritonavir on DYT-TOR1A MEF EV protein abundances, given that both drugs augment ISR activity (4, 25, 26). Ritonavir and salubrinal effects on the putative DYT-TOR1A biomarker proteins were positively correlated (Pearson *r* = 0.47, *p* < 0.0001) (Figure 3D). This result is consistent with a degree of shared mechanism of action between salubrinal and ritonavir.

In summary, ISR tool compound experiments demonstrate that ISR activity effects correlate with DYT-TOR1A genotype disruptions and ritonavir corrective effects on MEF EV protein composition. These results support the hypothesis that DYT-TOR1A genotype disruptions of MEF EV protein abundances and the corrective effects of ritonavir treatment are related, at least in part, to ISR pathway activity.

### Stratification of EV components for biomarker potential in human samples

In this study, we have taken advantage of the benefits of control over biological variables that an animal model system affords to generate initial proteomic discovery datasets for putative biomarkers of DYT-TOR1A. To guide translation to dystonia biomarker discovery in future patient-derived cell line or human plasma and CSF samples, we considered the results from our three experimental tests alongside human biospecimen datasets to prioritize candidates with the greatest potential.

Our stratification process considered the following features. First, we identified protein candidates that have been previously detected in human plasma (27). This criterion identified 164 of the 363 genotype-disrupted proteins. Second, we identified candidates that showed conserved directionality of effects across two drug perturbations, independent of effect size or *p*-value (Ritonavir-DYT, ISRIB-WT) (Supplementary Data Sheet S1 Columns 5,6). This criterion identified 121 of 164 proteins. Then, we created a composite score of genotype and ritonavir effect sizes by summing the absolute value of their respective Cohen’s *d* score. The results of this analysis are compiled in Supplementary Data Sheet S1. Overall, a third of the DYT-TOR1A genotype disrupted proteins show favorable characteristics according to these prioritizations.

## Discussion

Here we used a discovery proteomics approach to determine whether DYT-TOR1A alters EV composition by comparing EVs isolated from DYT-TOR1A heterozygous knockin MEF cultures to those from wildtype littermate controls. We identified a subset of 363 proteins with significant genotype effects. We then tested their pharmacodynamic responsivity to candidate drugs and found that ritonavir has a strikingly broad corrective effect on the EV proteome, and that at least a subset of these changes further correlates with ISR activity. Altogether, the results of this study provide preclinical proof-of-principle for the potential to use EVs in DYT-TOR1A for predictive and pharmacodynamic biomarker applications and define a prioritized list of candidate biomarkers based on follow-on testing and human bioinformatic data.

A significant takeaway from this exploratory study is that in DYT-TOR1A, rather than identifying one or a handful of candidate biomarkers, we found broad proteome-wide disruptions and corrections. We hypothesize at least three mechanisms that could cause the widespread EV composition disturbances we observed in DYT-TOR1A. First, in previous *in vitro* studies of DYT-TOR1A, patient-derived dermal fibroblasts exhibit secretion deficits through the ER-to-Golgi secretory pathway (20, 28), which regulates trafficking to a variety of intracellular locations prior to extracellular release (29). EVs are a heterogeneous population of vesicles produced by distinct biogenesis mechanisms—exosomes form as intraluminal vesicles within late endosomes and are released when these multivesicular bodies fuse with the plasma membrane, while microvesicles arise from direct outward budding of the plasma membrane (29–31). However, both carry cargo sorted and transported by the ER-to-Golgi pathway, and the ultracentrifugation EV isolation method used in this study likely includes a mixed EV population (32, 33). Broad-based changes in DYT-TOR1A EV composition may reflect upstream disruptions in these intracellular trafficking pathways. Second, ΔE-TorsinA abnormally localizes to the nuclear envelope relative to TorsinA’s usual predominance in the ER, and this mislocalization is likely to influence trafficking through the nuclear envelope (19, 34–37). A third mechanism that could cause broad EV compositional changes is the influence of ISR dysregulation on protein synthesis in DYT-TOR1A. ISR dysfunction is implicated in the pathogenesis of DYT-TOR1A and other dystonias (20). The ISR regulates mRNA translation at the level of translation initiation (38). ISR activity markedly and globally reconfigures which proteins are translated (38–40). In addition, HIV protease inhibitors (including ritonavir) activate the ISR (4, 26) and show corrective effects on several DYT-TOR1A phenotypes (4). Therefore, the influence of the ISR on global proteostasis could contribute to the EV proteome genotype effects and ritonavir effects we observed. Although EV cargo loading is a regulated process, rather than a simple stochastic loading of nearby proteins (31, 41), a sufficiently large change in proteostasis could be reflected across multiple subcellular compartments, including EVs. Future studies examining the intracellular dynamics of the DYT-TOR1A candidate biomarkers identified in this study could further test these three candidate pathophysiological mechanisms.

It was striking that the strongest and broadest drug effect identified in this study was caused by ritonavir and not ISR-targeting tool compounds. In measuring the pharmacodynamic responsivity of DYT-TOR1A genotype-disrupted proteins to ritonavir, we found that 63% of these proteins (230/363) were significantly different in DYT-TOR1A following ritonavir treatment and 95% of these changes were in the corrective direction toward WT. This is best illustrated by unsupervised hierarchical clustering of each sample showing that all three ritonavir-treated DYT-TOR1A samples cluster closer to WT cell lines than their DYT vehicle-treated corresponding cell lines. These results identify a proteomic “signature” that could be used as a measure of pharmacodynamic response.

Many of the differentially abundant EV proteins identified in this study show strong cross-species homology and are detected in human plasma. Since similar mouse-to-human predictive approaches have proven useful in other diseases (42), we aimed to generate a prioritized candidate biomarker set using the advantages of the mouse model system to guide pharmacodynamic biomarker discovery in human patients with this rare disease.

Identifying a DYT-TOR1A biomarker signature also has implications for other forms of dystonia beyond DYT-TOR1A that may benefit from predictive biomarkers. While DYT-TOR1A has a recognizable clinical manifestation and is readily diagnosed by genotype testing, sporadic dystonias with no known genetic etiology are the most common form of dystonia. We have previously shown that ~4% of sporadic cervical dystonia patients had mutations in ATF4, the main effector protein of the ISR, and several other inherited dystonias also have ISR involvement (20, 43–46). We therefore anticipate that EV biomarkers may be useful not only for pharmacodynamic monitoring but also for identifying dystonia subpopulations with shared pathophysiology. Such predictive biomarkers could help identify sporadic dystonia patients who are most likely to respond to ritonavir or other ISR-modifying treatments in future clinical trials. Finally, a common but poorly understood feature of many inherited dystonias is that they show reduced penetrance. Current DYT-TOR1A genetic mouse models are not suited to address whether EV biomarkers may also have prognostic value because the model does not reproduce the dystonia phenotype. Future human studies will be needed to determine whether DYT-TOR1A EV biomarkers vary based on symptom manifestation and can be used to predict disease penetrance. Our results provide proof-of-concept that DYT-TOR1A genotype disrupts EV composition and its pharmacodynamic responsiveness under the more optimal homogenous conditions afforded by mouse models. We hope that these findings will accelerate future biomarker discovery.

## Materials and methods

### Experimental blinding, power, and statistical approach

Sample size was arbitrarily set *a priori* at three samples per group. Experimenters were blinded to MEF cell line genotype and drug treatment prior to cell culture experiments, and proteomics were performed on these blinded sample groups. Experimenters were unblinded after initial differential abundance analyses were completed. For differences in protein abundances, statistical testing used unpaired Student’s t tests between *n* = 3 WT and *n* = 3 DYT samples without correction for multiple hypothesis testing or with Bonferroni correction where noted (23). Two-tailed binomial sign test was performed using a null probability *p* = 0.5. Fisher’s Exact Test was performed on contingency tables for overlaps of the 363 significantly different proteins between conditions using abundances greater than or less than zero log_2_ fold change.

### Animals

ΔE Torsin1a knockin (courtesy of Dr. W. Dauer, UTSW; IMSR_JAX:025637) (19) mice on C57BL/6 background were bred in standard housing conditions with food and water provided *ad libitum*. All procedures were approved by the Duke University Institutional Animal Care and Use Committee (IACUC).

### Cell lines and cell culture

Mouse embryonic fibroblasts were harvested as previously described (37) from E14 *TOR1A*^ΔE/+^ mice and immortalized *via* SV40 transfection. MEFs were maintained in sterile-filtered MEF media [DMEM (Thermo Fisher Scientific, #11995–065) + 10% fetal bovine serum (Hyclone, #SH0071.03) + 1X GlutaMAX (Gibco, #35050–061) + 1% penicillin/streptomycin/amphotericin (Gibco, #15240062) + 1% Non-Essential Amino Acids (Gibco, #11140050)+ 55 nM β-Mercaptoethanol (Gibco, #21985023)] at 37°C/5% CO_2_.

### MEF EV-conditioned media collection

EV-depleted (dEV) media was prepared by spinning 10% FBS MEF media for 18 h at 100,000 × g (Beckman L8–55M ultracentrifuge; SW27 rotor; 23,600 rpm; 4°C) (47). MEFs were seeded at 5.8 × 10^5^ cells into one 15 cm dish per line. At 90% confluence, cells were passaged 1:10 into four 15 cm dishes per line and when each line reached ~50% confluence, media was exchanged for dEV media containing 1% dEV FBS and the given drug treatment. Ritonavir (Tocris Biosciences, #5856), ISRIB (Sigma, #SML0843), and salubrinal (Tocris Biosciences, #2347) were dissolved in DMSO (100 mg/mL) and frozen in aliquots at −20°C. On the day of each treatment, these aliquots were thawed and added to dEV media containing 1% FBS to final concentrations (0.04% DMSO vehicle, 50 nM ISRIB, 20 μM ritonavir, or 20 μM salubrinal). After 24 h in dEV media, the EV-conditioned media and cells were collected separately.

### EV protein isolation

EV-conditioned media was centrifuged at 4°C for 20 min at 2000 × g (Sorvall HS-4, 3500 rpm). Supernatant was transferred to a new tube and centrifuged at 4°C for 30 min at 8000 × g (Sorvall HS-4, 6500 rpm). Final clarified supernatant was stored at −80°*C.* Media was thawed in room temperature water bath and 36 mL per sample was ultra-centrifuged in Ultra-Clear tubes (Beckman Coulter, #344058) for 16 h at 100,000 × g (Beckman L8–55M ultracentrifuge; SW27 rotor; 23,600 rpm; 4°C) to isolate EVs (21). The supernatant was discarded and protein was extracted from the pellet. Protein was extracted by adding 50 μL modified RIPA buffer [1% Triton X-100, 0.5% SDS, 0.5% deoxycholic acid, 50 mM NaPO_4_ at pH 7.4, 150 mM NaCl, 2 mM EDTA, 50 mM NaF, 10 mM sodium pyrophosphate, 1 mM sodium orthovanadate, and protease inhibitor cocktail (Roche, #04693159001)], vortexing on low speed for 15 s, and shaking on an orbital shaker for 1 h at 4°C. Cell lysates were prepared in 1 mL modified RIPA buffer by rotating on a Nutator for 2 h. Lysates were then sonicated and centrifuged for 10 min at 10,000 × g to remove insoluble material, and the supernatant was taken as the whole cell lysate protein fraction.

### Immunoblotting

Total EV protein concentrations were quantified using a Micro BCA^™^ Protein Assay Kit (Thermo Fisher Scientific, #23235) and cell lysate protein concentrations were quantified by Pierce^™^ BCA assay (Thermo Fisher Scientific, #23225). Proteins were resolved on 4%–15% TGX gels (BioRad, #5671085), transferred to nitrocellulose membrane, blocked in TBS-T (0.1% Tween-20) with 5% BSA, and probed as indicated. Densitometry was quantified using ImageJ (48). The following primary antibodies and dilution ratios were used for immunoblotting experiments: anti-Actin—1:5000 (Millipore, #MAB1501); anti-TSG101—1:1000 (Abcam, #ab30871); anti-calnexin—1:1000 (Proteintech, #10427–2-AP).

### Quantitative mass spectrometry proteomics

#### Sample Preparation:

The Duke Proteomics and Metabolomics Core Facility (DPMCF) received 18 samples (3 biological replicates each of six conditions). Methods are as described in (37) with minor modifications and restated here for convenience: “Samples were first normalized to 20 μg and spiked with undigested casein at a total of 40, 80, or 160 fmol/μg, then reduced with 10 mM dithiolthreitol for 30 min at 80°C and alkylated with 20 mM iodoacetamide for 30 min at room temperature. Next, they were supplemented with a final concentration of 1.2% phosphoric acid and 741 μL of S-Trap (Protifi) binding buffer (90% MeOH/100 mM triethylammonium bicarbonate). Proteins were trapped on the S-Trap, digested using 20 ng/μL sequencing grade trypsin (Promega) for 1 h at 47°C, and eluted using 50 mM triethylammonium bicarbonate, followed by 0.2% formic acid, and lastly using 50% acetonitrile/0.2% formic acid All samples were then lyophilized to dryness and resuspended in 40 μL 1% trifluoracetic acid/2% acetonitrile containing 12.5 fmol/μL yeast alcohol dehydrogenase (ADH_YEAST). A Sample Pool QC (SPQC) was created from 3 uL of each sample. SPQCs were run periodically throughout the acquisition period.

#### Quantitative Analysis Methods:

Quantitative LC-MS/MS was performed on 2 μL of each sample, using a nanoAcquity UPLC system (Waters Corp) coupled to a Thermo Orbitrap Fusion Lumos high resolution accurate mass tandem mass spectrometer (Thermo) *via* a nano-electrospray ionization source. Briefly, the sample was first trapped on a Symmetry C18 20 mm × 180 μm trapping column (5 μL/min at 99.9/0.1 v/v water/acetonitrile), after which the analytical separation was performed using a 1.8 μm Acquity HSS T3 C18 75 μm × 250 mm column (Waters Corp.) with a 90-min linear gradient of 5%–30% acetonitrile with 0.1% formic acid at a flow rate of 400 nL/min with a column temperature of 55°C. Data collection on the Fusion Lumos mass spectrometer was performed in a data-dependent acquisition (DDA) mode of acquisition with a r = 120,000 (at m/z 200) full MS scan from m/z 375 – 1500 with a target automatic gain control (AGC) value of 2e5 ions. MS/MS scans were acquired at Rapid scan rate (Ion Trap) with an AGC target of 5e3 ions and a max injection time of 25 m. The total cycle time between full MS scans was 2 s. A 20 s dynamic exclusion was employed to increase depth of coverage.

#### Proteomics Data Analysis:

Following 22 total UPLC-MS/MS analyses (including 4 SPQC injections) were imported into Proteome Discoverer 2.3 (Thermo Scientific Inc.), and analyses were aligned based on the accurate mass and retention time of detected ions (“features”) using Minora Feature Detector algorithm in Proteome Discoverer. Relative peptide abundance was calculated based on area-under-the-curve (AUC) of the selected ion chromatograms of the aligned features across all runs. The MS/MS data was searched against the SwissProt *M. musculus* database, SwissProt *bovine* database (downloaded Sept 2019) and an equal number of reversed sequence “decoys” for false discovery rate determination. Mascot Distiller and Mascot Server (v 2.5, Matrix Sciences) were utilized to produce fragment ion spectra and to perform the database searches. Database search parameters included fixed modification on Cys (carbamidomethyl) and variable modifications on Meth (oxidation) and Asn and Gln (deamidation). Full trypsin enzyme rules were selected with 2 ppm precursor and 0.8 Da product ion mass tolerances. Peptide Validator and Protein FDR Validator nodes in Proteome Discoverer were used to annotate the data at a maximum 1% protein false discovery rate.

Following data alignment and AUC quantitation, missing values were imputed in the following manner. If less than half of the values are missing within any one treatment group, values are imputed with an intensity derived from a normal distribution defined by measured values within the same intensity range (20 bins). If greater than half values are missing for a peptide in a group and a peptide intensity is > 5e6, then it was concluded that peptide was misaligned and its measured intensity is set to 0. All remaining missing values are imputed with the lowest 5% of all detected values. These data were then subjected to a sample loading normalization in which the total signals were summed and those summed values were used as normalizing factors across all samples. All peptide AUCs belonging to the same protein were then summed together to generate a protein level intensity” (37).

Data were analyzed using GraphPad Prism v9 and R v4.2.0. Hierarchical clustering was performed in R using Euclidean distance measures and average-linkage clustering (49).

### Potential candidate biomarker criteria

For proteins, our DYT-TOR1A genotype-dependent subset of 363 differential proteins were annotated as “In Human Plasma” based on their presence in a public database, the Human Plasma Proteome Project (HPPP) (50, 51). The HPPP is a set of >3500 proteins that have been detected with varying degrees of evidence in different mass spectrometry studies. We focused on HPPP proteins that were detected in a minimum of 3 distinct studies. This criterion identified 164 of the 363 genotype-disrupted proteins.

We next used Cohen’s *d* as a standardized effect size for each genotype and drug treatment condition. This was calculated using the formulas below (52), where *n*_1_ and n_2_ are group sample sizes, *s*_1_ and s_2_ are group standard deviations, and *s*^2^_pooled_ is a pooled variance calculated using both groups’ features.

$$Cohen^{\prime }s\;d=\frac{{\overset{‾}{X}}_{\mathrm{Exp.}}-{\overset{‾}{X}}_{\mathrm{Control}\;}}{s_{\mathrm{pooled}}}$$

$$s_{\mathrm{pooled}\;}^{2}=\sqrt{\frac{\left(n_{1}\;-\;1\right){s_{1}}^{2}\;+\;\left(n_{2}\;-\;1\right){s_{2}}^{2}}{n_{1}\;+\;n_{2}\;-\;2}}$$

To rank candidate proteins by their genotype and ritonavir-treatment effect sizes, the absolute value of Cohen’s *d* for each condition was summed to make a combined score, “Absolute Cohen’s *d* Sum (Geno+RTV).” Candidate biomarkers were filtered based on the directionality of their pharmacodynamic response to ritonavir and ISRIB being concordant with genotype predictions (ritonavir opposing genotype directionality, ISRIB reproducing genotype directionality). These criteria were then combined to stratify biomarker subsets as displayed in Supplemental Data Sheet S1.

## Supplementary Material

Supplementary Material 2

Supplementary Material 1

The Supplementary Material for this article can be found online at: https://www.frontierspartnerships.org/articles/10.3389/dyst.2023.11053/full#supplementary-material.

## Data Availability

All raw data and Protein Discoverer results files that support this study are publicly available in MassIVE.ucsd.edu under the identifier MSV000090835.
