# Supplementary material for: DYT-TOR1A genotype alters extracellular vesicle composition in murine cell model and shows potential for biomarker discovery
Source: Dystonia. Author manuscript; Available in PMC 2026 Aug 22. (PMC13496261; doi:10.3389/dyst.2023.11053)
Supplement: Supplementary file 1 [file NIHMS2198721-supplement-Supplementary_Material_1.pdf]

Supplemental Figures

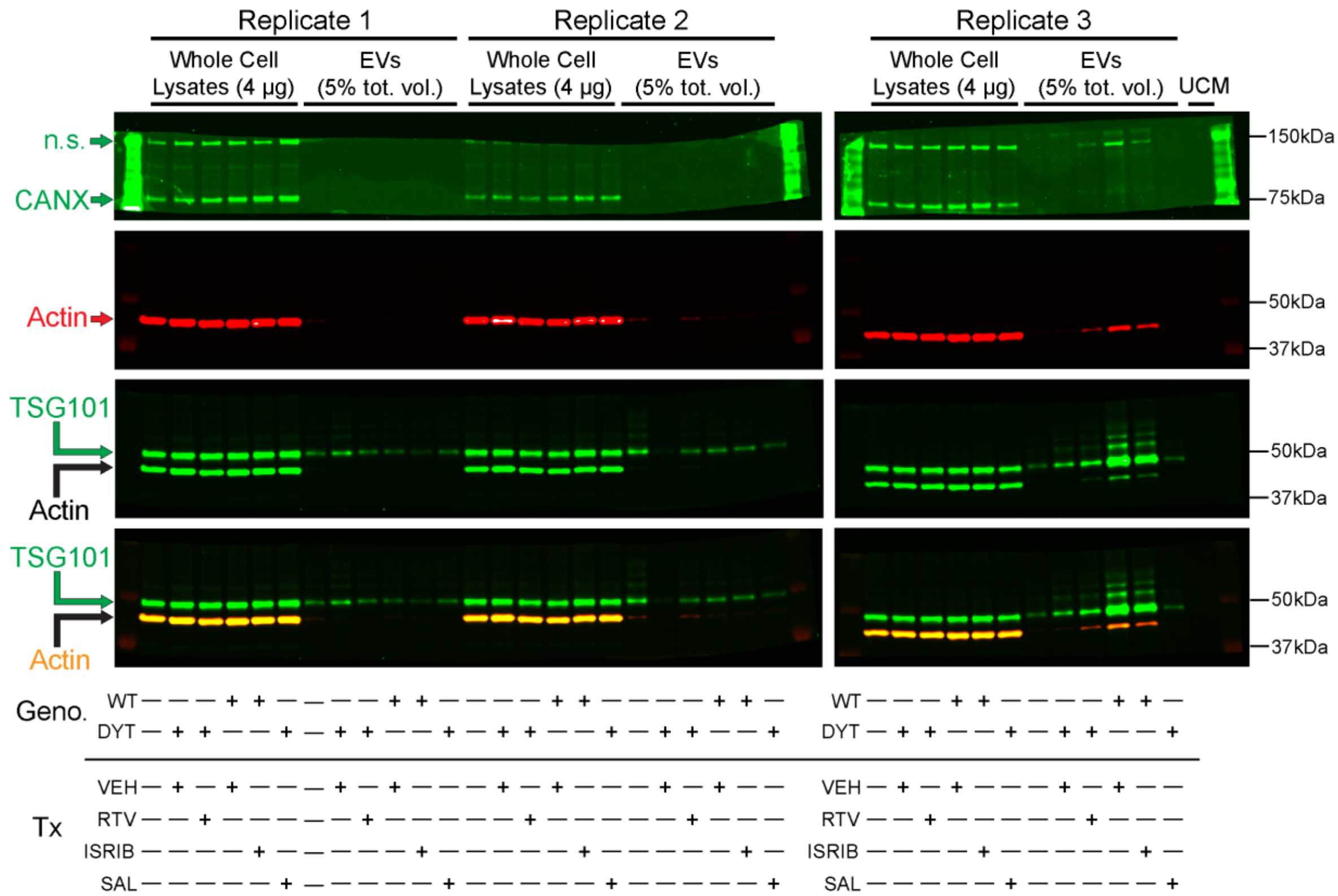

Figure S1. Non-cropped Western blots for samples in Figure 1D. Blinded samples were loaded onto gels by EV preparation batches. Unconditioned media (UCM) is the 'pellet' material recovered from ultracentrifugation of 1% dEV FBS unconditioned media as a negative control processed in parallel with conditioned media EV samples. Blots were probed for TSG101 (EV marker), calnexin (ER marker), and actin. Rows 2-4 show the same blots probed for both mouse anti-actin and rabbit anti-TSG101. Row 4 is shown with both red (700nm) and green (800nm) channels to demonstrate specificity of multiplexed primary and secondary antibodies. "n.s." indicates non-specific bands.

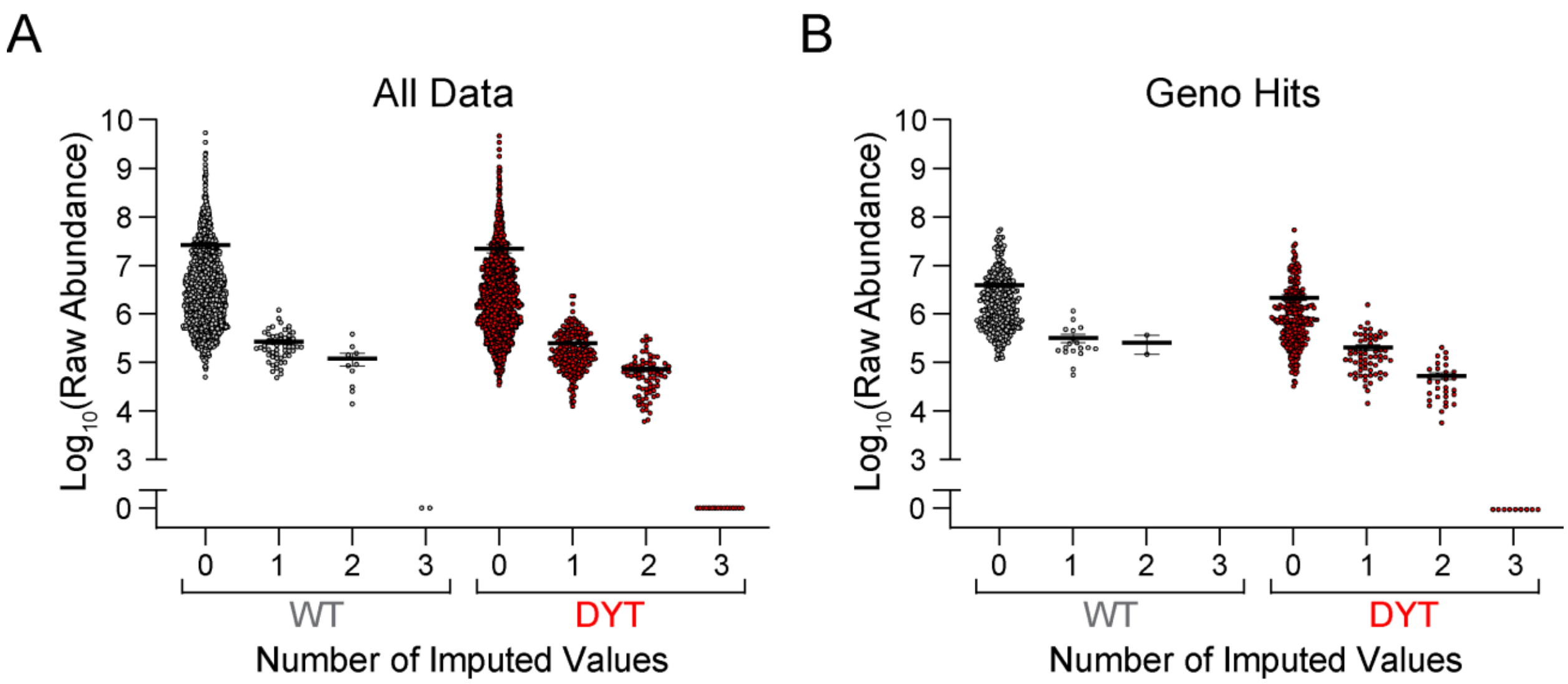

Figure S2. Presence of imputed values in LC-MS/MS raw abundance data from Figure 1F. EV proteins from vehicle-treated WT or DYT EV samples are grouped by number of missing (and imputed) values prior to normalization for **(A)** all data (n=1974) or **(B)** the subset of DYT genotype-dependent hits (n=363). (see Methods for imputation paradigm). "0 imputed values" indicates proteins were detected in all WT or DYT samples. 1, 2, or 3 imputed values show proteins not detected in the respective number of samples in each condition.
